# Comparative Proteomic Analysis of the Striatum in Heterozygous and Null DAT Knockout Rats

**DOI:** 10.3390/ijms27177758

**Published:** 2026-08-29

**Authors:** Alexey G. Mittenberg, Anastasia D. Belskaya, Sergey V. Shabelnikov, Zoya S. Fesenko, Polina A. Sylko, Raul R. Gainetdinov, Anna B. Volnova, Anastasia N. Vaganova

**Affiliations:** 1 Institute of Cytology of the Russian Academy of Sciences, Tikhoretskiy Prospekt, 4, 194064 St. Petersburg, Russia; mittenberg@incras.ru (A.G.M.);; 2 Institute of Translational Biomedicine, St. Petersburg State University, Universitetskaya Nab. 7/9, 199034 St. Petersburg, Russiast101564@student.spbu.ru (P.A.S.);; 3 Center for Transgenesis and Genome Editing, St. Petersburg State University, Universitetskaya Nab. 7/9, 199034 St. Petersburg, Russia

**Keywords:** neurodevelopmental disorders, ADHD, proteome, DAT, ASD, dopamine, striatum

## Abstract

Deregulation of striatal neurotransmission is a key pathogenetic mechanism in neurodevelopmental disorders such as attention deficit hyperactivity disorder (ADHD) and autism. In the present study, we applied a proteomic approach to demonstrate shifts in striatal protein expression in rats with heterozygous (DAT-Het) and homozygous (DAT-KO) dopamine transporter (DAT) gene knockouts. These animals model dose-dependent ADHD- and autism-like behaviors, ranging from slightly increased activity and social disturbances in DAT-Het rats to a pronounced phenotype in DAT-KO rats. We revealed pronounced changes in the proteomic profiles of both groups, associated primarily with deregulation of proteins involved in energy and carbon metabolism. Furthermore, we identified changes in vesicular transport proteins specific to DAT-KO and DAT-Het rats. Since these changes involved SNARE complex components, we evaluated SNARE mRNA expression in our models and public transcriptomic data for mouse models of neurodevelopmental disorders, including *Mbd5* gene haploinsufficiency and a polygenic model of ADHD. No significant changes in mRNA levels were revealed in any model. Thus, the identified protein expression changes likely depend on post-transcriptional mechanisms. These data suggest a deregulation of metabolism in DAT-Het rats, which becomes more pronounced in DAT-KO rats.

## 1. Introduction

The striatum plays an important role in decision-making and action selection, primarily based on glutamatergic inputs from the cortex, thalamus, and limbic regions, as well as dopaminergic afferents from the substantia nigra pars compacta (SNc) and ventral tegmental area (VTA) [1,2,3]. The main targets of cortical inputs in the striatum are GABAergic spiny projection neurons (SPNs), which represent 95% of the total neuronal population [3]. The remaining 5% of striatal neurons are regulatory interneurons, including cholinergic, fast-spiking, low-threshold-spiking, and several minor subtypes [3,4]. Both SPNs and striatal interneurons express different subtypes of DA receptors and receive dopaminergic innervation [5]. At the same time, around 80% of dopaminergic synapses are also associated with other presynaptic terminals in synaptic hub structures. These dopamine hub synapses are formed with cholinergic (14%), GABAergic (26%), and glutamatergic synapses (42%) [6].

Attention deficit hyperactivity disorder (ADHD) is one of the most common comorbid disorders of autism spectrum disorder (ASD) that can accompany autism [7]. Thus, up to 70% of individuals with ASD also present ADHD [8]. The recognition of this fact has led to the introduction of the term ‘AuDHD’ to describe people who have both autism and ADHD [9]. Currently, both diseases are considered as neurodevelopmental disorders, and their high comorbidity is suggested to be related to the common pathogenic mechanisms.

Dysregulation of dopaminergic activity in the striatum is a possible cause of a number of pathological conditions and neurodevelopmental disorders. In rodent models, methamphetamine self-administration [10], high alcohol preference [11], and antipsychotic administration [12] are associated with differentially expressed and phosphorylated synaptic components in the striatum. Neurodevelopmental disorders, including ADHD and ASD, are accompanied with altered volumes of the striatum and its subregions (e.g., the putamen, caudate and n. accumbens), as well as changes in the density of striatal neurons [13,14]. These alterations can cause an imbalance between sensorimotor and cognitive inputs, leading to social and motor impairments [15,16,17,18]. Furthermore, neurodevelopmental disorders are associated with altered connectivity between brain structures, including compromised structural integrity of corticostriatal tracts [14,19,20,21,22], aberrant striatum activity [23,24], and increased local functional connectivity between striatal subregions [25].

Clinical studies indicate that ADHD individuals may exhibit anomalous cross-regulation between cortical and striatal D2/3 receptors [26], while ASD subjects display increased DRD2 expression within the striatum [27]. A study of adult patients with ADHD found differences in cortical thickness that correlated with the severity of their symptoms [28].

Currently, there are a number of animal models of ASD that exhibit autistic-like behavior characteristics. All of these models are characterized by impaired social interactions, repetitive stereotypical behavior patterns, and memory and attention deficits [29]. Although animal models do not fully replicate the characteristics of ASD, some researchers have suggested that autistic behavior in humans may result from dysfunction in the midbrain dopaminergic system [30]. In particular, in the ASD model rats acquired by prenatal valproate exposure, a reduction in tyrosine hydroxylase (TH) content has been observed in the striatum. Despite TH being the key enzyme for dopamine synthesis, which is limiting for dopamine production, increased dopamine levels were found in the striatum and, as well, in the frontal cortex, cerebellum, and pons in rats after prenatal valproate exposure. By contrast, decreased dopamine levels were found in the hippocampus, midbrain, and serum of these animals [21,27]. 

Animal models of ADHD are also being actively developed to find new adequate approaches to the treatment of this disease [31]. It is believed that this syndrome is associated with impaired dopamine signaling in the striatum [32,33], with the dopamine reuptake transporter (DAT) playing a key role in this process [34,35]. Genetically modified animal models with impaired dopamine homeostasis mimic some of the symptoms of ADHD [36]. 

Dopamine transporter knockout (DAT-KO) mice [33,36] and rats [37] were created to investigate disorders rooted in disrupted dopamine transmission. The absence of DAT prevents dopamine reuptake, significantly prolonging dopamine release from the synaptic cleft and increasing extracellular dopamine levels in the striatum. Homozygous DAT-KO rats exhibit an approximately eight-fold elevation in extracellular striatal dopamine concentrations [37]. As a result, dopamine levels in the synaptic clefts of DAT-KO rats are increased, but the total dopamine concentration in the striatum, midbrain, and spinal cord tissues is reduced, and dysregulation of serotonin neurotransmission has also been evidenced [38].

As a result of these abnormalities in monoamine signaling, DAT-KO rats exhibit a distinct phenotype characterized by pronounced motor hyperactivity with attentional deficits [35,37,39,40] (as seen in ADHD patients), stereotypy and cognitive rigidity [22,41] (characteristic of ASD), sexual behavioral disturbances [42], and the manifestation of asocial-like autistic phenotypes [43]. Animals also demonstrated impairments in learning and spatial memory [44], and involuntary attention (prepulse inhibition and sensorimotor filtering) [37,45].

Rats with a deficiency of the DAT gene can be considered a model of neurodevelopmental disorders, exhibiting specific behavioral and neurobiological features that align with ADHD and autism spectrum disorders. Additionally, these rats display reduced body size and weight, along with postural alterations, relative to their WT littermates. Thus, DAT-KO animals serve as models of specific behavioral phenotypes that mimic the characteristics of ADHD and ASD. At the same time, given the established links between DAT dysfunction and neuropsychiatric pathology, DAT-KO animals, including mice and rats, serve as translational models for neurodevelopmental disorders.

Of particular note are heterozygous (DAT-Het) littermates, who retain one functional allele and exhibit milder physiological abnormalities [28] but still have severe impairments in social behavior [43].

Proteomic analysis of synaptosomes isolated from the striatum of DAT-KO rats and their wild-type littermates previously revealed the decreased relative abundance of several proteins, including proteins involved in axonal–dendritic transport, postsynaptic modulation of chemical synaptic transmission, and regulation of receptor-mediated endocytosis [46]. The present study investigates the global, whole-tissue striatal proteome, which was not described previously in the DAT-KO rats. Furthermore, we expand the experimental design by introducing DAT-heterozygous rats to capture intermediate disease phenotypes and uncover novel homeostatic adaptations. DAT-Het rats exhibit intermediate molecular changes in the striatum and other brain regions [47,48,49], accompanied by significantly less pronounced behavioral disturbances compared to their homozygous DAT-KO counterparts. 

Considering the previously described molecular and functional disturbances in the striatum of dopamine transporter knockout rats, this study aims to characterize the proteomic changes in this structure in homo- (DAT-KO) and heterozygous (DAT-Het) DAT knockout rats. Taking into account that the striatum represents the primary anatomical site for DAT expression and signaling, the molecular alterations and compensatory mechanisms triggered by DAT dysfunction will compromise the overall function of this structure, ultimately leading to subsequent downstream impairments and distinct phenotypic manifestations. Therefore, we performed the profiling of the striatal proteome to improve the understanding the molecular background of DAT knockout-related features in rats. To capture global alterations in the striatal proteome, an untargeted label-free proteomic screening of pooled striatal lysates was performed using LC-MALDI analysis. Next, we quantitatively measured the expression of target mRNAs in striatal tissue obtained from homozygous and heterozygous DAT knockout rats using RT-PCR.

## 2. Results

### 2.1. LC-MALDI Proteomic Profiling of DAT-Deficient Rats’ Striatum

**Figure 1:**
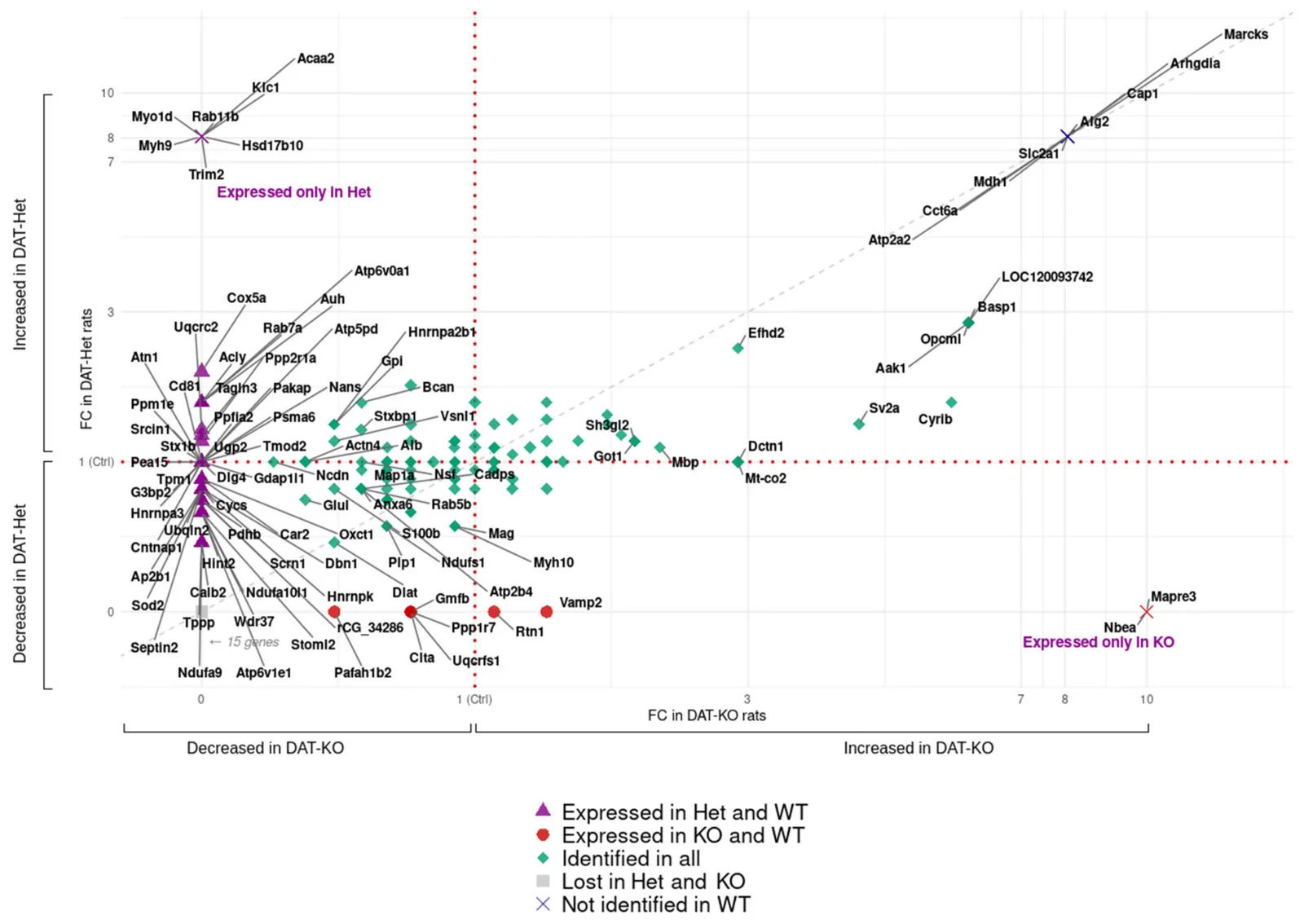
Semi-quantitative analysis of proteomic data in DAT knockout rats. Relative expression levels in heterozygous (Het) and homozygous (KO) DAT knockout rats are presented as fold change (FC) compared to WT. An FC of 1 (i.e., the same level as in WT) is marked by red dotted lines. Proteins that are over- or under-represented by a factor of two or more compared to WT (FC > 2 or FC < 0.5) are indicated.

The peptide samples fractionated on a nano-LC system (Eksigent NanoLC Ultra 2D+ system, Sciex, Darmstadt, Germany) were analyzed with an AB Sciex 5800 TOF/TOF instrument operated in the positive ion mode. The LC-MALDI analysis of striatal lysates yielded a diverse set of proteins with distinct distribution profiles across the experimental groups (Figure 1).

We identified 186 proteins in striatal lysate, including 15 unique proteins for WT rats, 2 unique proteins for DAT-KO rats, and 8 unique proteins for DAT-Het rats. Additionally, seven proteins were identified in both DAT-Het and DAT-KO rats but were not revealed in WT rats (Supplementary Table S1). Thus, only 97 proteins were identified mutually; 169, 163, and 117 were identified in WT, DAT-Het, and DAT-KO rats, respectively. Of note, the semi-quantitative assay did not reveal a gradual decrease in protein content and diversity in DAT-Het and DAT-KO rats compared to WT samples. Some proteins, which were not identified in DAT-KO rats, seem to be overproduced in DAT-Het samples compared to WT.

### 2.2. KEGG Pathway Enrichment Analysis of Differentially Expressed Proteins in DAT Knockout Rats

**Figure 2:**
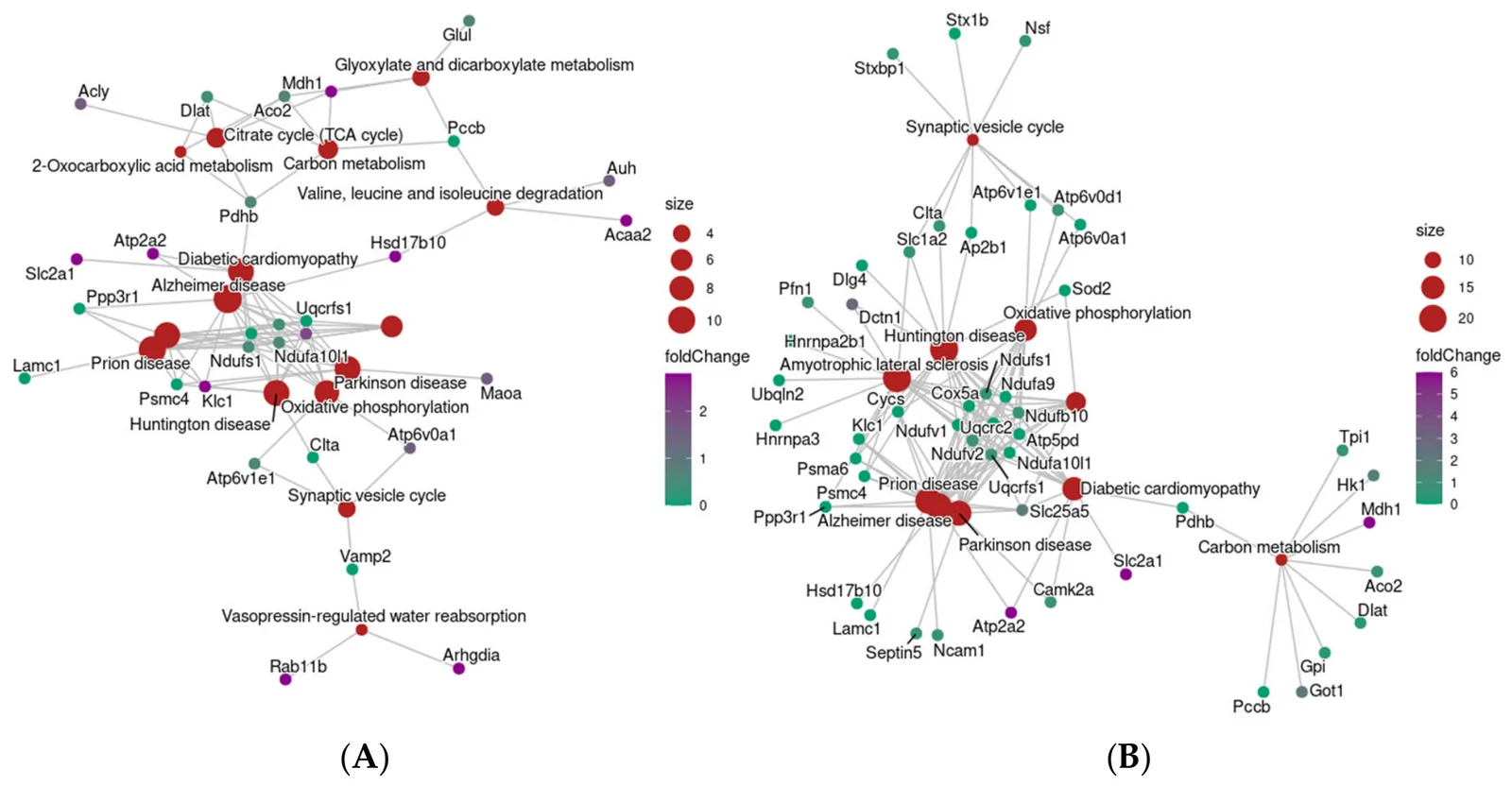
KEGG pathway enrichment analysis of proteins exhibiting altered expression in DAT-Het (**A**) and DAT-KO (**B**) rats compared to their WT littermates.

To expand the number of enriched terms, we included all proteins whose expression levels were up- or downregulated by at least 1.5-fold in DAT-Het or DAT-KO rats compared to their WT littermates. Considering that pathway dysregulation may involve the downregulation of some gene products accompanied by an increase in the expression of other pathway components, we included both upregulated and downregulated proteins simultaneously (Figure 2A,B).

The enrichment results were highly concordant between DAT-Het and DAT-KO rats. Specifically, we identified widespread dysregulation of energy and glucose metabolism, accompanied by changes in the expression of several proteins involved in nervous system function, such as Lamc1, Ncam1, Slc1a2, and others. Such combinations of differentially expressed proteins resulted in the significant enrichment of these clusters with components related to neurodegenerative disease-specific KEGG pathways. Because the number of significantly downregulated proteins was higher in DAT-KO samples, the enrichment results were more complex in this group (Figure 2B) compared to the DAT-Het rats (Figure 2A). In both studied genotypes, the enrichment cores of the identified pathways consisted predominantly of downregulated proteins.

One of the most neuron-specific expression shifts observed in the DAT-KO and DAT-Het groups involved the synaptic vesicle cycle pathway, particularly within the machinery regulating exocytosis and vesicle interaction with the presynaptic membrane, including SNARE components. DAT-Het striatal samples were characterized by a decreased level of Vamp2 protein, which is a part of this complex, and in DAT-KO rats, another part of this complex, Stx1b, was decreased. Additionally, Stxbp1, which interacts with Stx1b, was also decreased in DAT-KO rats’ striatal samples. Thus, we additionally performed a targeted analysis of the mRNA expression of genes associated with the SNARE complex, as described below. The mapping of enrichment results for differently expressed proteins in DAT-KO and DAT-Het striatum samples on the synaptic vesicle cycle KEGG pathway is represented in Supplementary Figure S1.

### 2.3. SNARE Complex Gene mRNA Expression in the Striatum in DAT-KO Rats

**Figure 3:**
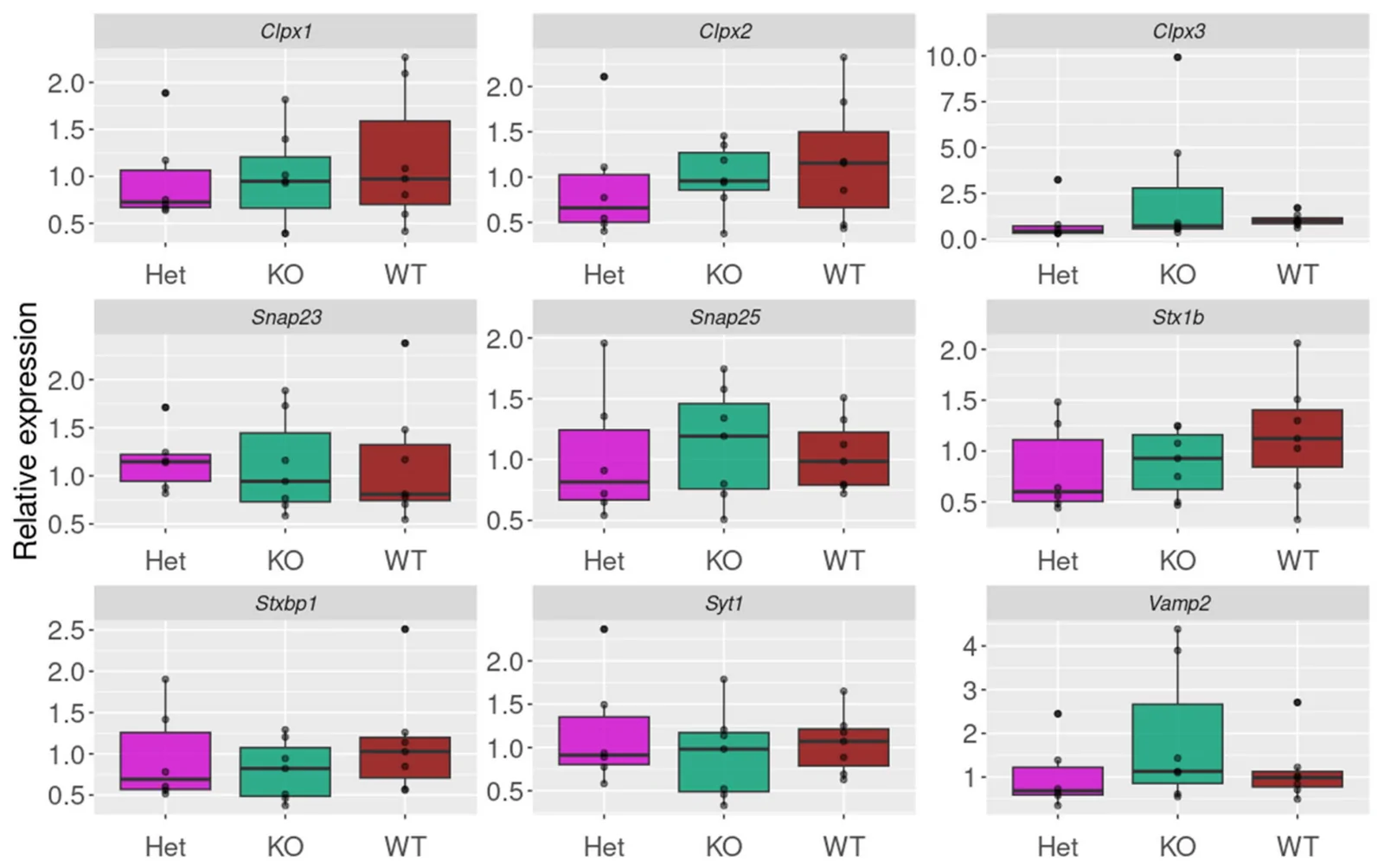
Expression of SNARE complex genes in the striatum of DAT knockout rats. Relative mRNA expression levels in wild-type (WT), heterozygous (Het), and homozygous (KO) DAT knockout rats are presented as 2^−ΔΔCt^-normalized values.

To evaluate the transcriptional regulation of the SNARE complex, we quantified the mRNA expression of *Stx1b*, *Stxbp1*, *Snap25*, *Snap23*, *Vamp2*, *Cplx1*, *Cplx2*, *Cplx3*, and *Syt1* genes in striatal tissue harvested from the DAT knockout rat line using RT-qPCR. No significant differences in expression levels were observed among wild-type (WT), heterozygous (DAT-Het), and homozygous (DAT-KO) rats (Figure 3).

### 2.4. GEO Data Search and Analysis

An initial search of the GEO database [44] retrieved 79 entries, from which four GEO SuperSeries records were excluded. Following a rigorous filtering process to eliminate non-relevant or incomplete datasets—such as single-cell RNA sequencing data, *in vitro* studies, non-striatal samples, small sample sizes (n < 5 per experimental group), or a lack of behavioral validation confirming relevance to ADHD and/or ASD—six candidate reports were retained for further evaluation. Of these, four datasets were subsequently excluded due to the unavailability of raw count tables. Ultimately, two specific RNA-seq datasets (explicitly listed in Table 1) met all criteria and were selected for downstream comparative analysis.

**Figure 4:**
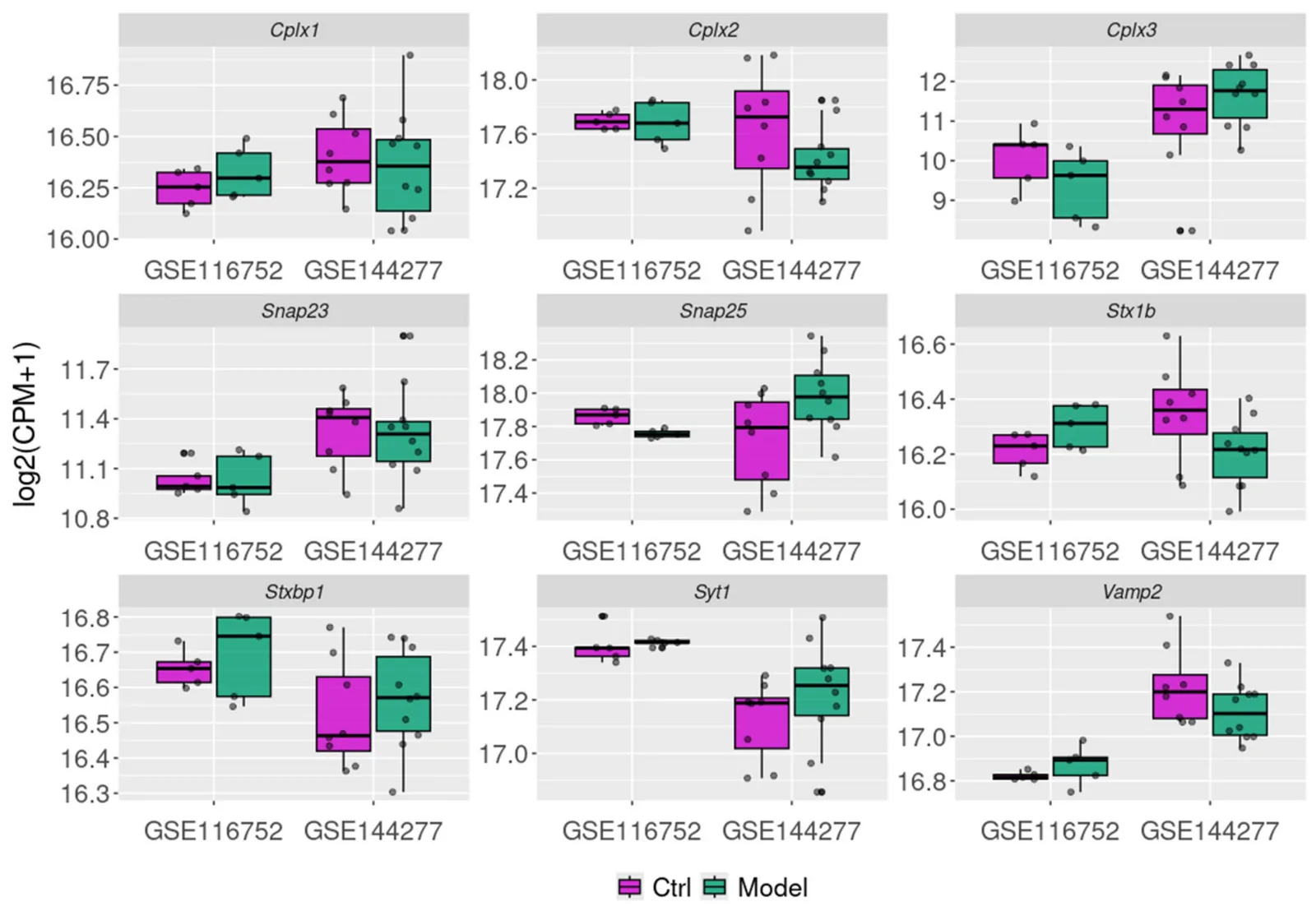
Expression of SNARE complex genes in mouse striatum samples represented in GEO116752 and GSE144277 GEO datasets. CPM—count per million.

Consistent with the RT-qPCR data from the DAT knockout rats, no significant differences were observed in the expression of *Stx1b*, *Stxbp1*, *Snap25*, *Snap23*, *Vamp2*, *Cplx1*, *Cplx2*, *Cplx3*, and *Syt1* genes in these animal models of neurodevelopmental disorders compared to their respective wild-type littermates (Figure 4).

## 3. Discussion

Recently, several specific features of striatal morphology and function were revealed in DAT-KO rats, which are considered a model for ADHD and manifestations of other neurodevelopmental and psychiatric diseases in humans. These rats demonstrate a pronounced volume loss in the dorsal striatum, pronounced hyperconnectivity in the cortico-striatal circuit, and an increase in extracellular dopamine concentrations accompanied by a decrease in total dopamine content [46,48]. In the present study, we evaluated the changes in the proteomic profile of both DAT-KO rats with pronounced behavioral disturbances and DAT-Het rats, which demonstrate mild hyperactivity and social abnormalities under experimental conditions. These data expand the previous study, which estimated the synaptosomal proteome in DAT-KO rats and revealed the decreased relative abundances of proteins involved in axo-dendritic transport, postsynaptic modulation of chemical synaptic transmission, and regulation of receptor-mediated endocytosis [46].

As expected, the alterations observed in DAT-KO rats were more pronounced compared to those in their DAT-Het littermates. Striatal samples from DAT-KO rats lost the expression of significantly more proteins compared to WT rats. At the same time, in DAT-Het rats, the expression of several proteins was upregulated or downregulated rather than completely lost compared to WT. Interestingly, 17 proteins that were not identified in WT rats were revealed in the DAT-insufficient littermates (Figure 1).

Both DAT-haploinsufficient and DAT-KO rats demonstrate changes in the expression of proteins related to neurodegenerative diseases. These associations were revealed by KEGG pathway enrichment analysis and depend on the deregulation of basic energetic mitochondrial metabolism, accompanied by expression changes in several proteins involved in neurotransmitter signaling. Among the latter is the glial transporter Slc1a2, which clears glutamate from the extracellular space. Its expression increases in both knockout variants. At the same time, monoamine oxidase A (Maoa), which metabolizes dopamine and regulates its levels [50], is downregulated in both DAT-Het and DAT-KO animals in our study.

The primary striatal proteomic differences identified between WT rats and DAT-Het/DAT-KO rats are heavily associated with mitochondrial function and carbon metabolism. The principal role of mitochondria is energy production via the oxidative phosphorylation (OXPHOS) pathway. Within the brain, neurons largely rely on OXPHOS for ATP production, whereas astrocytes preferentially depend on glycolysis [51]. Beyond energy supply, mitochondria are critically involved in reactive oxygen species (ROS) production, intracellular Ca^2+^ homeostasis, and apoptosis; consequently, mitochondrial dysfunction encompasses aberrant energy metabolism, oxidative damage, and compromised lipid metabolism [51]. 

Despite differences in their core symptoms and diagnostic criteria, ASD and ADHD frequently co-occur, sharing underlying metabolic endophenotypes. For instance, mitochondrial DNA copy number (mtDNA-cn) has been proposed as a complex biomarker reflecting mitochondrial activity, which is predominantly under nuclear genetic control [52,53]. While approximately 5% of children with ASD are diagnosed with classic mitochondrial disease, a significantly larger proportion exhibits subclinical mitochondrial dysfunction [54,55]. Taken together, these insights reinforce the notion that neurodevelopmental pathologies are closely linked to neuronal dysfunction arising from oxidative stress, mitochondrial impairment [56], and the dysregulation of cerebral glucose metabolism [57].

We also revealed differences in the expression of synaptic vesicle machinery proteins in DAT-deficient rats. In both genotypes, several proteins involved in this process were markedly decreased or completely lost (i.e., became undetectable using the applied method). However, the identified proteomic profiles were genotype-specific. Stx1b and Stxbp1, which are involved in SNARE complex functioning, were downregulated in DAT-KO rats. At the same time, in DAT-Het rats, the disturbances in this pathway were milder; however, these rats demonstrated a loss of Vamp2 expression, which, like Stx1b, is part of the SNARE complex.

The SNARE complex is the core machinery driving synaptic vesicle exocytosis. It consists of SNAP-25, vesicle-associated membrane proteins (VAMPs), and syntaxins, alongside key regulatory elements such as synaptotagmins (SYTs) [58]. For instance, complexins stabilize the core SNARE bundle in a highly fusogenic state, maintaining a primed vesicle pool primed for rapid, synchronous release upon calcium influx sensed by SYT [58,59,60,61]. Furthermore, Syntaxin 1A (STX1A) is essential for anchoring presynaptic membrane fusion, while the entire complex is subsequently disassembled by the NSF ATPase and its cofactor, SNAP, to allow vesicle recycling [62]. Notably, across various ASD and ADHD models, the expression levels of several vesicular transport proteins, including SNAP-25, STX1A, STX1B, and VAMP2, are significantly altered [63,64,65].

Studies on postmortem human samples and various animal models of neurodevelopmental disorders consistently demonstrate that genes governing synapse maintenance and neurotransmission are dysregulated under these conditions. Specifically, prominent changes in SNARE abundance have been documented in striatal tissue [58]. The identified decrease in expression in SNARE complex components and associated protein Stxbp1 may be interpreted as an adaptation for the hyperdopaminergic striatum state in these animals. However, it also may be related to the decrease in general neurotransmission activity in the striatum, mirroring the gradual decompensation of the striatal function in DAT-Het and DAT-KO rats.

In animal models of neurodevelopmental disorders, a discrepancy between the protein and gene expression data in brain structures suggests post-transcriptional or post-translational modifications [66]. At the same time, the identified discrepancy between qPCR and proteomics data may be related to the complex nature of striatal samples, which consist of both striatal neurons and projections from distant brain structures, such as the substantia nigra or motor cortex. Thus, the identified differences in SNARE protein profiles, as well as other differences between WT, DAT-KO, and DAT-Het rats, may be related to gene transcription deregulation outside the striatum.

The presented results should be interpreted within the context of several study limitations. 

While this discovery-based approach allowed for a broad mapping of the proteome, it relies on data that can be influenced by ionization efficiency rather than absolute protein concentration. Consequently, the observed changes reflect relative differences in protein expression across genotypes rather than exact absolute quantities, necessitating subsequent targeted validation for specific candidates. Second, the semi-quantitative screening in this study was performed using a pooled sample strategy, which limits the application of stringent statistics. Therefore, our analysis focuses on identifying robust, large-scale trends and co-regulated pathways rather than individual biological variance and single-protein shifts. Another limitation of the method is the preferential detection of highly abundant proteins; data regarding low-abundance receptors or upstream regulators that initiate downstream signaling pathways and physiological processes may be under-represented due to low protein abundance, insufficient signal quality, poor peptide ionization, limited MS coverage, or other technical factors. As well, our analysis included proteins exhibiting relatively subtle expression changes (less than a 2-fold change) in DAT knockout animals relative to controls; notably, RT-qPCR may lack the sensitivity required to robustly detect such minor fluctuations at the mRNA level. Simultaneously, data regarding post-translational modifications are incomplete in the present study and were omitted from the global analysis. Nevertheless, critical proteins mediating synaptic transmission are known to undergo S-nitrosylation in the striatum, including VAMP2/synaptobrevin, SNAP-25, CPLX1, and SYT1. For instance, CPLX1 undergoes S-nitrosylation, which induces functional and synaptic localization changes that may alter its interaction with the SNARE complex [64], alongside other modifications. Finally, we focused specifically on the vesicular transport alterations as the neuron-specific process, deliberately leaving the complex shifts in systemic metabolic regulation outside the scope of the present study.

Despite certain limitations, our findings demonstrated highly consistent trends across both DAT knockout groups. These results indicate that the model effectively recapitulates neurodevelopmental disorders at the molecular level. Furthermore, the identified changes in proteins critical for vesicular transport and energy metabolism warrant further investigation, offering potential biomarkers to assess therapeutic efficacy in translational studies utilizing this model.

## 4. Materials and Methods

### 4.1. Animals

Striatal samples were collected from 5-month-old male rats and categorized into three genotypes: homozygous DAT knockout (DAT-KO, n = 5 for proteomic studies and n = 7 for RT-PCR), heterozygous DAT knockout (DAT-Het, n = 5 for proteomic studies and n = 7 for RT-PCR), and wild-type controls (WT, n = 5 for proteomic studies and n = 7 for RT-PCR). The DAT-KO line was derived by mating heterozygous parents, with genotypes verified according to an established protocol. Animals were maintained in individually ventilated cages (IVC; RAIR IsoSystem World Cage 500, Lab Products, Inc., Seaford, DE, USA) under standard conditions: a 12 h light/dark cycle (lights on at 09:00), a 22 ± 1.0 °C ambient temperature, and 50–70% relative humidity. Food and water were provided ad libitum.

For sampling, rats were humanely sacrificed by decapitation under deep anesthesia induced by 1% isoflurane in 100% oxygen (Chemical Iberica Produktos Veterinarios, Salamanca, Spain). For proteomic studies, dissected striatal tissues were immediately placed into liquid nitrogen for protein preservation. For RT-PCR, samples were immediately stored in ExtractRNA solution (Evrogern, Moscow, Russia). All animal experiments adhered to ethical regulations and received approval from the Institutional Animal Care and Use Committee of Saint Petersburg State University (protocol no. 131-03-6, 25 April 2025).

### 4.2. Sample Preparation and Label-Free LC-MALDI Mass Spectrometry

Whole-tissue striatum tissue samples were homogenized on ice using a Silent Crusher S homogenizer (Heidolph Instruments, Schwabach, Germany) in a lysis buffer containing 10 M urea, 2% CHAPS, 0.5 mM phenylmethylsulfonyl fluoride (PMSF), 0.01× protease inhibitor cocktail cOmplete™ Protease Inhibitor Cocktail (Roche Diagnostics, Mannheim, Germany), and 1% DeStreak reagent (Cytiva, Marlborough, MA, USA). The resulting homogenates were subjected to ultrasonication.

To evaluate sample quality, aliquots of the protein extracts were analyzed via SDS-PAGE according to the Laemmli method. Protein aliquots were mixed with a 4× loading buffer (0.5 M Tris-HCl, pH 6.8, 2% SDS) and rhodamine dye, incubated for 10 min at 37 °C, and loaded onto a 10% polyacrylamide gel. Electrophoresis was performed in a Tris-glycine running buffer (25 mM Tris, 192 mM glycine, 0.1% SDS, pH 8.3) using a Mini-PROTEAN Tetra Cell system (Bio-Rad Laboratories, Hercules, CA, USA). Gels were stained with Coomassie Brilliant Blue G-250 and subsequently destained with 7% acetic acid. Protein molecular weights were estimated using pre-stained protein markers (10–180 kDa, SolarBio Science & Technology, Beijing, China). Protein quantification was normalized against a known concentration of the protein marker.

Protein samples were solubilized in 8 M urea. Proteins were reduced with 10 mM DTT for 30 min, alkylated with 25 mM IAA for 45 min in the dark at room temperature, and then digested with modified trypsin (Trypsin Gold, Promega, Madison, WI, USA). Overnight digestion was performed at 37 °C at a specific enzyme-to-protein ratio, ensuring the final urea concentration was below 1 M. The resulting peptides were desalted and concentrated usin Strata-X 30 mg solid-phase extraction tubes (Phenomenex, Torrance, CA, USA). Eluted fractions were supplemented with 10 μL of 50 mg/mL D-glucose to aid subsequent rehydration and dried in a rotor vacuum evaporator (Martin Christ, Osterode am Harz, Germany). Then, digests were resuspended in 25 μL of 1% (*v*/*v*) aqueous formic acid. Following filtration through a 0.2 μm PVDF filter, peptide concentrations for mass spectrometry were determined by UV extinction at 205 and 280 nm. 

Chromatographic separation was achieved on a nano-LC system (Eksigent NanoLC Ultra 2D+, Sciex, Darmstadt, Germany) equipped with a Chromolith CapRod RP-18e HR reversed-phase column (0.1 mm × 150 mm, Merck, Darmstadt, Germany). A total peptide load of 1000 ng was resolved using a 44 min linear gradient of 6.5–43% mobile phase B at a flow rate of 400 nL·min^−1^. Mobile phases consisted of A (water with 0.2% *v*/*v* TFA) and B (80% *v*/*v* acetonitrile in water). Effluent was continuously mixed at 2.4 μL·min^−1^ with a matrix solution containing 9 mg·mL^−1^ CHCA in 85% acetonitrile and 0.2% (*v*/*v*) TFA, supplemented with two calibration standards, bradykinin 2–9 (30 pM·mL^−1^) and ACTH 18–39 (60 pM·mL^−1^), at a flow rate of 2.4 μL·min^−1^. A micro-fraction collector deposited 1 mm spots every 2 s, yielding a 44 × 16 array of 704 targets per run. Prior to subsequent injections, the column was washed with a gradient (0–100–100% B for 5 min and 2 min, respectively, at a flow rate of 600 nL·min^−1^) and equilibrated to 0% B for 3.5 min before subsequent injections.

Targeted plates were analyzed using a TOF/TOF 5800 System (Sciex) in positive ion mode with the MALDI stage set to continuous motion. MS spectra were acquired at a laser intensity of 3300 (500 shots/spectrum; 250 shots/sub-spectrum). For MS/MS analysis, the intensity was increased to 4600 using a DynamicExit algorithm with a high spectral quality threshold, capped at 1000 shots/spectrum (250 shots/sub-spectrum). Up to 25 top precursor ions per spot within a mass range of 750–3500 Da and an S/N > 40 were selected for fragmentation.

Protein Pilot 5.0.1 software (Sciex, Darmstadt, Germany) with the Paragon algorithm in thorough mode was used for the MS/MS spectra search against the Uniprot rat database. Carbamidomethyl cysteine was set as a fixed modification. False discovery rate (FDR) analysis was performed by analyzing reversed sequences using the embedded PSEP tool, with the threshold for strictly confident protein and peptide identification set at FDR < 1%. The MS/MS data was converted to mzidentml format for further analysis using Scaffold 4.0 software. Protein relative abundance was determined using an untargeted, label-free intensity-based quantification strategy. Relative protein expression levels across the experimental samples (DAT-KO, DAT-Het, and WT) were calculated based on the summation of precursor peptide ion intensities (total ion current, TIC) extracted by Scaffold 4.0 software. To account for systematic run-to-run variations and slight differences in sample loading, total intensity normalization was applied across all mass spectrometry runs. 

All samples were analyzed in technical triplicate. Proteins were included in the final list of accepted identifications only if they were detected in all replicates of at least one experimental group. Protein abundance differences between the DAT-deficient lineages and the WT control group were subsequently expressed as fold change (FC) values. Due to the sample pooling strategy, missing value imputation was not performed; proteins undetected in a specific group pooling were treated as below the limit of detection, with unique proteins requiring at least 2 unique peptides for confident identification. The mass spectrometry proteomics data have been deposited at Mendeley Data, V1, https://doi.org/10.17632/6tsdm23n2y.1.

### 4.3. Functional Enrichment and KEGG Pathway Analysis

All proteins whose expression levels were up- or downregulated by at least 1.5-fold in DAT-Het or DAT-KO rats compared to their WT littermates were considered differentially expressed.

Kyoto Encyclopedia of Genes and Genomes (KEGG) pathway enrichment analysis was performed on clusters of proteins whose expression levels were up- or downregulated by at least 1.5-fold in DAT-Het or DAT-KO rats compared to their WT littermates. Enrichment analysis and visualization of the results were performed using the clusterProfiler (v.4.6.0) R package [67], which is designed to conduct over-representation analysis (ORA) to determine which a priori-defined gene sets are significantly enriched in the data. The enrichKEGG function with default arguments was applied, using all genes listed in the database as the background. A cnetplot visualization within clusterProfiler was used to represent the enrichment networks.

### 4.4. Gene Expression Omnibus Database Search

The RNAseq data were mined from the public database GEO from NCBI [68] by the search request ("neostriatum"[MeSH Terms] OR "corpus striatum"[MeSH Terms] OR Striatum[All Fields]) AND ((((((((ASD[All Fields] OR ("autistic disorder"[MeSH Terms] OR autism[All Fields])) OR ("autistic disorder"[MeSH Terms] OR autistic[All Fields])) OR ("attention deficit disorder with hyperactivity"[MeSH Terms] OR ADHD[All Fields])) OR ("attention"[MeSH Terms] OR attention[All Fields])) OR hyperactivity[All Fields]) OR neurodevelopmental[All Fields]) OR neurodevelopment[All Fields]) AND "Expression profiling by high throughput sequencing"[Filter] AND ("10"[n_samples] : "10000"[n_samples])) AND ("Expression profiling by high throughput sequencing"[Filter] AND ("10"[n_samples] : "10000"[n_samples])) in the GEO DataSets repository. The inclusion criteria were as follows: (1) at least 5 striatal samples per study subgroup in the dataset, including intact WT samples; (2) the datasets represent the expression profiles of native samples, and data for single-cell RNA sequencing or cell fractions were excluded; (3) clear description of behavioral abnormalities matching ASD or ADHD phenotypes in the dataset description or associated papers; and (4) raw count data are available.

### 4.5. Transcriptomic Data Analysis

Data from the GEO repository were obtained as raw counts, which were utilized for differential expression analysis (DEA) and subsequently converted to counts per million (CPM) for visualization.

DEA was performed using the quasi-likelihood (QL) F-test framework in the edgeR (v. 4.6.2) R package [69]. Low-expressed genes were removed prior to analysis using the filterByExpr function with default parameters. Library sizes were normalized using the calcNormFactors function via the trimmed mean of M-values (TMM) method. *p*-values were adjusted using the Benjamini–Hochberg procedure, and genes with a false discovery rate (FDR) < 0.05 were identified as differentially expressed genes (DEGs).

### 4.6. RNA Extraction, Reverse Transcription, and qPCR Analysis

Total RNA was isolated from striatal specimens using ExtractRNA reagent (Evrogen, Moscow, Russia) according to the manufacturer’s guidelines. A NanoDrop 2000 spectrophotometer (Thermo Scientific, Waltham, MA, USA) was employed to evaluate RNA concentration and purity.

Next, 300 ng of total RNA from each sample was converted into complementary DNA (cDNA). Reverse transcription was executed with the MMLV Reverse Transcriptase kit (Evrogen, Moscow, Russia) under recommended conditions.

Table 2 lists the gene-specific primers used in this work. Real-time amplification was performed on a CFX96 Touch Real-Time PCR Detection System (Bio-Rad, Hercules, CA, USA) in at least two independent runs per cDNA sample. Transcript levels of ND1–ND6 were determined using the qPCRmix-HS SYBR master mix (Evrogen, Moscow, Russia). The qPCR program consisted of 40 cycles of denaturation at 95 °C for 10 s, annealing at 60 °C for 15 s, and elongation at 72 °C for 30 s with simultaneous fluorescence data collection. Amplicons were subjected to melting curve analysis using the default instrument settings to verify the specificity of the PCR products.

### 4.7. Statistical Analysis

Relative gene expression levels were quantified utilizing the 2^−ΔΔCt^ methodology. The process commenced with the acquisition of cycle threshold (Ct) values for every sample. Subsequently, ΔCt values were established by subtracting the Ct of the internal control gene (Hprt) from that of the gene of interest. To obtain ΔΔCt, the average Ct value for each gene within the wild-type cohort was then subtracted from these ΔCt values. The ultimate relative expression levels were then derived by calculating 2^−ΔΔCt^.

Each experimental run was duplicated, and the mean values were employed for subsequent statistical processing. Prior to comparative analysis, data distribution normality was verified using the Shapiro–Wilk test. Discrepancies in normalized expression were analyzed either by a Student’s *t*-test (for data conforming to a normal distribution with *p* > 0.05 across all groups) or a Mann–Whitney U test, incorporating a Holm–Bonferroni correction for multiple comparisons. Outlier data points, identified through the interquartile range (IQR) criterion, were excluded from further examination.

## 5. Conclusions

In the present study, protein expression profiles were compared in striatal samples derived from rats with different doses of DAT gene knockout, which is suggested to be a model for neurodevelopmental disorders. In both DAT-deficient groups, i.e., DAT haploinsufficiency or null DAT knockout, basic carbohydrate and energy metabolism seems to be deregulated in the striatum compared to their WT littermates. These results align with current literature data, pointing out that energy and carbohydrate metabolism is impaired in ADHD and ASD subjects, as well as in rodent models of neurodevelopmental conditions. Expectedly, the deregulation pattern was more pronounced in homozygous DAT-KO rats than in DAT-Het animals. Additionally, a specific downregulation of proteins involved in synaptic vesicular transport was revealed in DAT-deficient rats. Considering the previously described decrease in SNARE complex-related proteins in the striatum of patients with neurodevelopmental disorders and relevant animal models and the high expression of proteins Syn1 and Stxbp1 identified in DAT-deficient rats, we additionally estimated mRNA expression for genes involved in SNARE functioning in DAT-Het and DAT-KO rats, as well as in public transcriptomic data. However, we did not identify any significant changes in the mRNA expression for these proteins. These results do not contradict the proteomic results, because a plethora of processes in neural tissues are regulated on a post-transcriptional level. Thus, we demonstrate that in DAT-deficient rats representing models of neurodevelopmental conditions, the energy metabolism of striatal tissue seems to be impaired, while the more pronounced phenotype in DAT-KO rats is also associated with the suppression of synaptic transmission. These features may be considered as biomarkers for further translational studies.

## Figures and Tables

**Table 1:** RNAseq datasets included in the analysis.

GEO ID	Title	Platform	Sample	Model
GEO116752	Striatal transcriptome of a mouse model of ADHD reveals a pattern of synaptic remodeling	Illumina HiSeq 2500	5 control and 5 model animals, 6.5 months old, male	To model ADHD polygenically, a mouse line was generated through selective breeding for high home-cage activity. Relative to controls, these high-active mice display hyperactivity and motor impulsivity that are successfully reversed by amphetamine administration [11].
GSE144277	Functional genomic consequences of MBD5 knockdown in mouse brain and CRISPR-derived neurons [mouse]	Illumina HiSeq 2000	8 control and 10 model animals, 8 weeks old, male and female	The *Mbd5* gene encodes methyl-CpG-binding domain protein 5, which is structurally associated with ASD; its heterozygous deficiency in mice (Mbd5^+/GT^ causes craniofacial dysmorphism, cognitive disabilities, impaired motor functions, and aberrant social behaviors [44].

**Table 2:** Primers and probes were used for quantitative RT-PCR in DAT-KO and DAT-Het rat study.

Target	Forward Primer	Reverse Primer
*Cplx1*	ATG CGG CAG GGC ATA CGA GAT A	TGC CTT CTG AGT TGG CCT CCA T
*Cplx2*	TGA GAA GGT CCG GCA GCA GAT T	CAG TGG CCC CGG CAG ATA TTT G
*Cplx3*	ACA AGG GGG ACG GAG ACA AGT C	CCC TGA AGT GAC TCC GTA GCG T
*Vamp2*	CCC TGC ACC TCC TCC AAA TC	GAT AGC TTC TGG TCC CGC TC
*Snap23*	GCA ACA TGG GGT GAT GGT GGA G	AGG ATG CTG CCC ACT TGA GTC A
*Snap25*	GTG GCT TCA TCC GCA GGG TAA C	GAG GTT TCC GAT GAT GCC GCT C
*Stx1b*	CCG AGG CTG CAT CGA GAA ACT G	CCG GAC CTT GTT TGC CGT CTT T
*Stxbp1*	AAC AAA CTC ATC CAG CAC GCC C	GCG TAG TGT GGA ATC CGT GAC G
*Syt1*	ACG CTG TCC CGA AAG TTT GTG G	GCA AAA CCC TGG TGA TGG CTG T
*rStx1a*	CGT AGC GGA GGG AGA AGC AGA T	TCC AAC TCC TCA CTG GTC GTG G
*Hprt*	CTC ATG GAC TGA TTA TGG ACA GGA C	GCA GGT CAG CAA AGA ACT TAT AGC C

## Data Availability

The mass spectrometry proteomics data have been deposited at Mendeley Data, V1, https://doi.org/10.17632/6tsdm23n2y.1, and Zenodo, https://doi.org/10.5281/zenodo.21873293.

## Supplementary Materials

The following supporting information can be downloaded at: https://www.mdpi.com/article/10.3390/ijms27177758/s1.
