# Supplementary material for: Comparative Proteomic Analysis of the Striatum in Heterozygous and Null DAT Knockout Rats
Source: Int J Mol Sci. 2026 Aug 29;27(17):7758. doi: 10.3390/ijms27177758 (PMC13566312; doi:10.3390/ijms27177758)
Supplement: Supplementary file 1 [file ijms-27-07758-s001.zip › Figure S1.pdf]

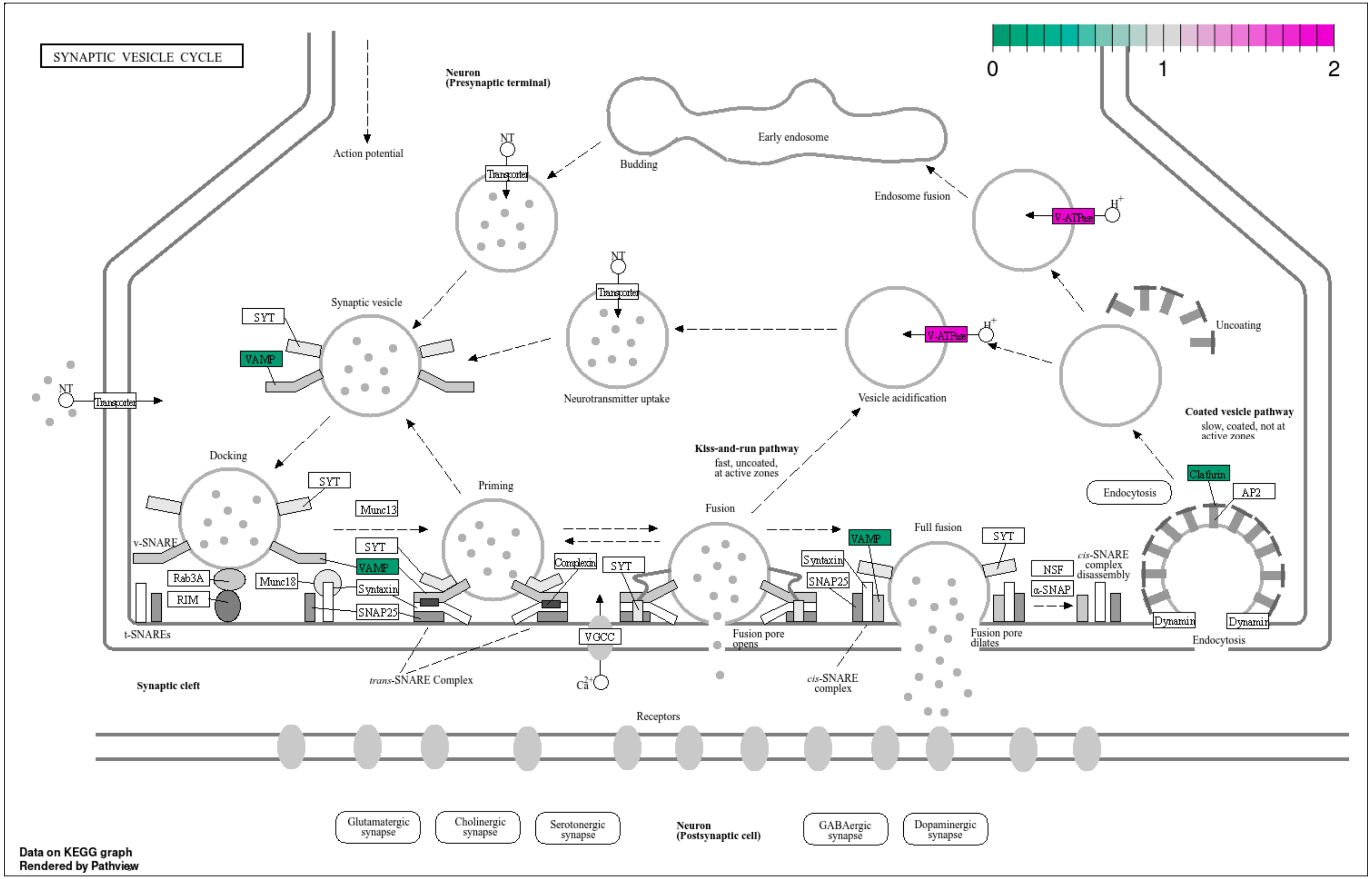
SYNAPTIC VESICLE CYCLE
0
1
2
Neuron
(Presynaptic terminal)
Action potential
Early endosome
Budding
Endosome fusion
NT
Transporter
V-ATPase
H+
Synaptic vesicle
SYT
VAMP
Neurotransmitter uptake
Vesicle acidification
Uncoating
Coated vesicle pathway
slow, coated, not at active zones
NT
Transporter
Docking
SYT
Munc13
Priming
Kiss-and-run pathway
fast, uncoated, at active zones
Fusion
Full fusion
Endocytosis
Clathrin
AP2
v-SNARE
Rab3A
RIM
Munc18
Syntaxin
SNAP25
Complexin
SYT
VAMP
NSF
α-SNAP
cis-SNARE complex disassembly
t-SNAREs
VGCC
Fusion pore opens
Fusion pore dilates
Dynamin
Dynamin
Endocytosis
Synaptic cleft
trans-SNARE Complex
Ca2+
cis-SNARE complex
Receptors
Glutamatergic synapse
Cholinergic synapse
Serotonergic synapse
Neuron
(Postsynaptic cell)
GABAergic synapse
Dopaminergic synapse
Data on KEGG graph
Rendered by Pathview

(a)

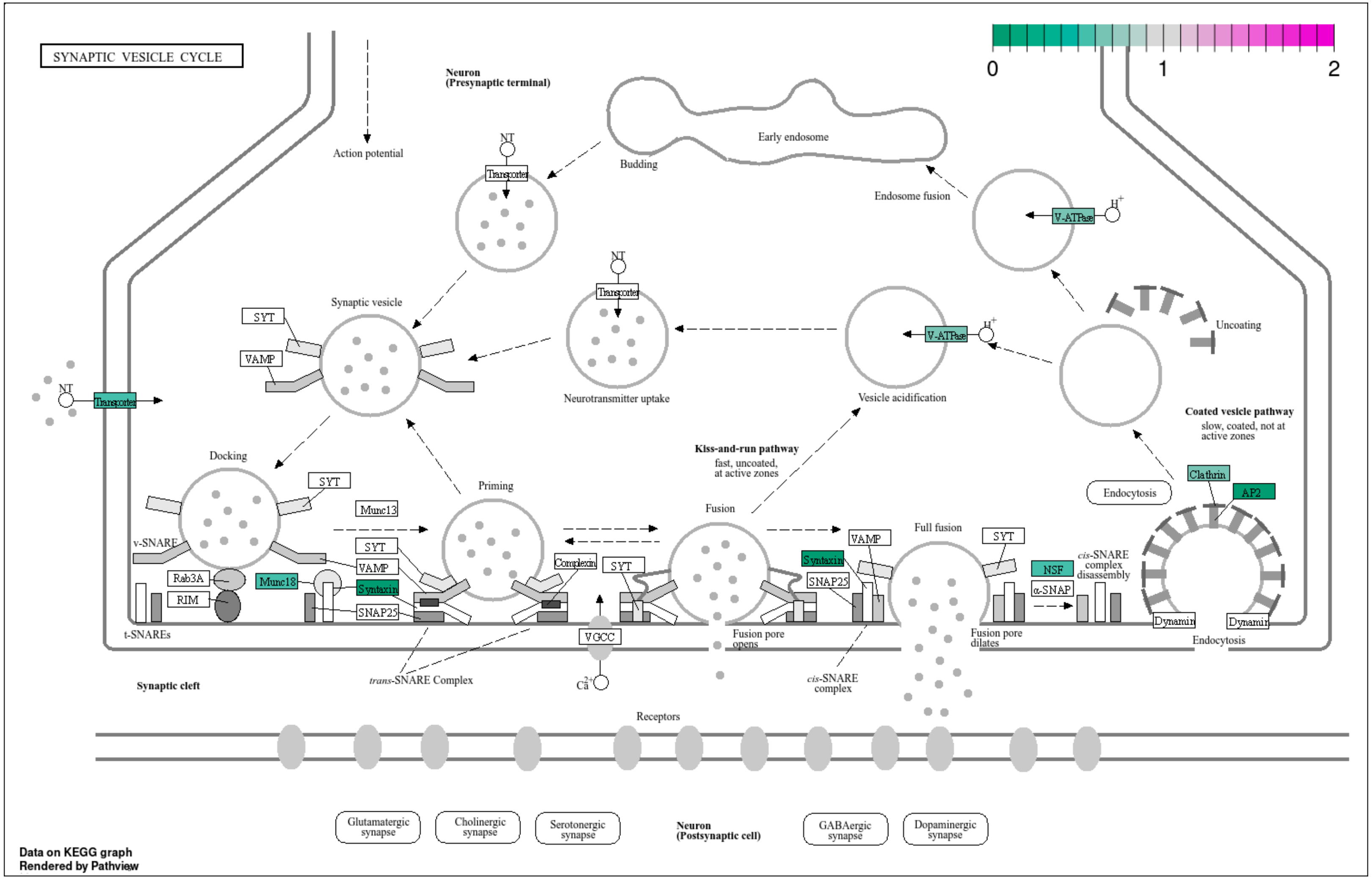

(b)

Figure S1. Expression pattern of proteins involved in the synaptic vesicle exocytosis KEGG pathway in DAT-Het (a) and DAT-KO (b) rats compared to their WT littermates. Downregulated and upregulated proteins are shown in green and magenta, respectively.
