# Supplementary material for: Comparative Proteomic Analysis of the Striatum in Heterozygous and Null DAT Knockout Rats
Source: Int J Mol Sci. 2026 Aug 29;27(17):7758. doi: 10.3390/ijms27177758 (PMC13566312; doi:10.3390/ijms27177758)
Supplement: Supplementary file 1 [file ijms-27-07758-s001.zip › Table S1.pdf]

Supplementary Table S1: List of identified proteins in the striatum of DAT-Het and DAT-KO rats with corresponding fold-change values compared to WT littermates.

Gene ID	Fold change in DAT-KO	Fold change in DAT-Het
AABR07051450	0.6	0.6
Aak1	Identified	Identified
Abat	0.7	0.8
Acaa2	Not identified	Identified
Acly	Not identified	2.1
Aco2	0.6	0.6
Actg1	1.2	1.1
Actn4	0.3	1
Alb	0.3	1
Aldoa	1.2	0.8
Aldoc	1.1	0.9
Alg2	Identified	Identified
Ank2	1.1	1.6
Anxa3	Not identified	Not identified
AnxaIdentified	0.5	0.7
Ap2b1	Not identified	0.7
Apoe	1	1.4
Arhgdia	Identified	Identified
Atn1	Not identified	1
Atp2a2	Identified	Identified
Atp2b4	0.5	0.7
Atp5f1a	0.8	1
Atp5f1b	1.5	1
Atp5pd	Not identified	1
Atp6v0a1	Not identified	2.1
Atp6v0d1	0.7	1
Atp6v1a	0.9	0.9
Atp6v1b2	0.9	0.8
Atp6v1e1	Not identified	0.5
Auh	Not identified	2.1
Basp1	Identified	Identified
Bcan	0.5	2.1
Cadps	0.5	0.7
Calb2	Not identified	0.3
Camk2a	0.7	0.7
Cap1	Identified	Identified
Car14	Not identified	Not identified
Car2	Not identified	0.8
Cct6a	Identified	Identified
Cd81	Not identified	1
Ckb	1	0.9
Clta	0.7	Not identified
Cltc	1.4	1.3
Cmpk2	1.4	1
Cnp	1	0.7
Cntnap1	Not identified	1
Cox5a	Not identified	3.1
Crmp1	0.6	0.9
Crym	0.7	1.6
Cycs	Not identified	0.7
Cyrib	5.7	2.1
Dbn1	Not identified	0.7
Dctn1	2.9	1
Dlat	0.4	0.3
Dlg4	Not identified	1

Dnm1	1.1	1.2
Dnm1l	0.7	0.9
Dpysl2	1.2	0.7
Dync1h1	0.9	1.3
Efhd2	2.9	4.2
Ehbp1	Not identified	Not identified
Fkbp11	0.9	1.3
G3bp2	Not identified	1
Gapdh	0.9	1.2
Gda	1.8	1.8
Gdap1l1	Not identified	1
Gdi1	1.2	1.7
Glud1	1.1	0.9
Glul	0.3	0.6
Gmfb	0.7	Not identified
Gnb1	0.6	0.7
Got1	2	1.3
Gpi	0.4	1.6
Hba-a1	1	1.2
Hdhd2	Not identified	Not identified
Hint2	Not identified	0.5
Hist1h4b	1.4	1
Hk1	1.6	1.3
Hnrnpa2b1	0.4	1.6
Hnrnpa3	Not identified	1
Hnrnpk	Not identified	0.7
Hsd17b10	Not identified	Identified
Hspd1	1.1	1.2
Iars2	Not identified	Not identified
Igsf8	1.4	0.7
Kcnab2	Not identified	Not identified
Klc1	Not identified	Identified
Lamc1	Not identified	Not identified
Ldha	1.9	1.4
LOC120093742	Identified	Identified
Lsamp	Not identified	Not identified
Mag	0.9	0.4
Maoa	1.4	2.1
Map1a	0.5	0.9
Map1b	0.6	1
Map2	0.6	1
Mapre3	Identified	Not identified
Marcks	Identified	Identified
Mbp	2.2	1.2
Mdh1	Identified	Identified
Mdh2	0.9	0.7
Mt-co2	2.9	1
Myh10	0.9	0.4
Myh9	Not identified	Identified
Myo1d	Not identified	Identified
Nans	Not identified	1
Napb	0.7	1
Nbea	Identified	Not identified
Ncam1	0.6	1.2
Ncdn	0.2	1
Ndufa10l1	Not identified	0.5

Ndufa9	Not identified	0.3
Ndufb10	0.7	1
Ndufs1	0.6	0.4
Ndufv1	Not identified	Not identified
Ndufv2	0.7	1.3
Nsf	0.5	1
Opcml	Identified	Identified
Oxct1	Not identified	0.8
Pafah1b2	0.4	Not identified
Pakap	Not identified	1
Pcbp1	Not identified	Not identified
Pccb	Not identified	Not identified
Pdhb	Not identified	0.6
Pdia3	1	2.1
Pea15	Not identified	1
Pebp1	0.6	0.6
Pfkm	1.1	1
Pfn1	0.7	1
Phb1l2	1.4	1
Pkm	0.9	1
Plp1	0.6	0.4
Ppfia2	Not identified	1
Ppm1e	Not identified	1
Ppp1r7	0.7	Not identified
Ppp2r1a	Not identified	1.3
Ppp3r1	Not identified	Not identified
Prdx3	0.7	1
PsmaIdentified	Not identified	1
Psmc4	Not identified	Not identified
Pygb	0.9	1
Rab11b	Not identified	Identified
Rab5b	0.5	0.7
Rab7a	Not identified	2.1
rCG_3428Identified	Not identified	0.6
Rhoa	0.7	0.5
Rtn1	1.1	Not identified
S100b	0.4	0.7
Scrn1	Not identified	0.6
Septin2	Not identified	0.7
Septin5	0.7	1
Sh3gl2	2	1.3
Sirt2	0.9	0.8
Slc12a5	1.4	1
Slc1a2	0.6	0.9
Slc25a5	1.8	1.6
Slc25a51l1	Not identified	Not identified
Slc2a1	Identified	Identified
Snap91	1.4	1
Sod2	Not identified	0.7
Sptan1	0.9	1
Sptbn1	1.1	1
Sptbn2	0.7	2.6
Srcin1	Not identified	1
Stmn1	0.7	0.5
Stoml2	Not identified	0.5
Stx1b	Not identified	1

Stxbp1	0.5	1.5
Sv2a	4.3	1.6
Syt1	1.4	1.7
Tagln3	Not identified	1.4
Tmod2	Not identified	1
Tpi1	0.6	0.8
Tpm1	Not identified	1
Tppp	Not identified	0.3
Trim2	Not identified	Identified
Ubqln2	Not identified	0.7
Ugp2	Not identified	1
Uqcrc1	1.3	1.2
Uqcrc2	Not identified	1.5
Uqcrfs1	0.7	Not identified
Vamp2	1.4	Not identified
Vcl	Not identified	Not identified
Vdac1	0.9	1
Vsnl1	0.4	1.3
Wdr37	Not identified	0.5

Identified – identified only in DAT-KO or DAT-Het, was not revealed in WT rats.
Non identified – identified in WT rats, but not in DAT-KO or DAT-Het respectively
